# PIK3R1, SPNB2, and CRYAB as Potential Biomarkers for Patients with Diabetes and Developing Acute Myocardial Infarction

**DOI:** 10.1155/2021/2267736

**Published:** 2021-11-30

**Authors:** Yue Zheng, Yuheng Lang, Zhenchang Qi, Wenqing Gao, Xiaomin Hu, Tong Li

**Affiliations:** ^1^School of Medicine, Nankai University, Tianjin 300071, China; ^2^Nankai University Affiliated Third Center Hospital, No. 83, Jintang Road, Hedong District, Tianjin 300170, China; ^3^The Third Central Clinical College of Tianjin Medical University, Tianjin 300170, China; ^4^The Third Central Hospital of Tianjin, 83 Jintang Road, Hedong District, Tianjin 300170, China; ^5^Tianjin Key Laboratory of Extracorporeal Life Support for Critical Diseases, Tianjin, China; ^6^Institute of Hepatobiliary Disease, Tianjin, China

## Abstract

**Background:**

Young patients with type 2 diabetes mellitus (DM) and acute myocardial infarction (AMI) have high long-term all-cause and cardiovascular mortality rates. We aimed to investigate the differentially expressed genes (DEGs) that might be potential targets for DM patients with AMI.

**Methods:**

Gene datasets GSE775, GSE19322, and GSE97494 were meta-analyzed to obtain DEGs of the left ventricle myocardium in infarcted mice. Gene datasets including GSE3313, GSE10617, and GSE136948 were meta-analyzed to identify DEGs in diabetes mice. A Venn diagram was used to obtain the overlapping DEGs. KEGG and GO pathway analyses were performed, and hub genes were obtained. Pivotal miRNAs were predicted and validated using the miRNA dataset in GSE114695. To investigate the cardiac function of the screened genes, a MI mouse model was constructed; echocardiogram, qPCR, and ELISA of hub genes were performed; ELISA of hub genes in human blood samples was also utilized.

**Results:**

A total of 67 DEGs were identified, which may be potential biomarkers for patients with DM and AMI. GO and KEGG pathway analyses were performed, which were mainly enriched in response to organic cyclic compound and PI3K-Akt signaling pathway. The expression of PIK3R1 and SPNB2 increased in the MI group and was negatively correlated to left ventricular ejection fraction (LVEF), whereas that of CRYAB decreased and was positively correlated to LVEF. Patients with high CRYAB expression demonstrated a short hospital stay and the area under the curves of the three protein levels before and after treatment were 0.964, 0.982, and 0.918, suggesting that PIK3R1, SPNB2, and CRYAB may be diagnostic and prognostic biomarkers for the diabetes patients with AMI.

**Conclusion:**

The screened hub genes, PIK3R1, SPNB2, and CRYAB, were validated as credible molecular biomarkers and may provide a novel therapy for diabetic cardiac diseases with increased proteotoxic stress.

## 1. Introduction

Cardiovascular diseases are associated with considerable mortality and morbidity and acute myocardial infarction (AMI) is the leading cause of mortality in human [1]. In addition, the mortality of AMI has increased 5.6 times in the last three decades [2]. Indeed, a better understanding of AMI progression may help in the diagnosis and treatment, thus saving patients' lives.

Previous research reported that MI liberated hematopoietic stem and progenitor cells from bone marrow niches via sympathetic nervous system signaling. The progenitors then seeded the spleen, yielding a sustained boost in monocyte production, which promoted atherogenesis and therefore contributed to MI progression [3, 4]. Persistent impairment of endothelial vasomotor function in the conduit arterial segment and the resistance arteriole is related to atheromatous plaque progression in the infarct-related coronary arteries of ST-elevated myocardial infarction (STEMI) survivors [5]. Mildly abnormal baseline lipid levels are associated with an increased future risk of atherosclerotic cardiovascular disease events, particularly MI, whereas other levels of lipid variability are not [6, 7]. Therefore, studies on abnormal baseline lipid levels and atherosclerosis may decrease the mortality and morbidity of patients with diabetes mellitus (DM) and undergoing AMI at the same time.

Young patients with type 2 DM and MI have higher long-term all-cause and cardiovascular mortality and more than one-third of patients die within 10 years, emphasizing the need for more aggressive secondary prevention for these patients [6]. The European Society of Cardiology (ESC) algorithms can be used to determine AMI without ST-elevation in patients with DM [8]. In the IMPROVE-IT clinical trial, the benefit of adding ezetimibe to statins was enhanced in patients with DM and high-risk patients without DM [9]. Although these therapies might be helpful, patients with DM remain a high-risk population in whom identification of AMI is challenging and requires careful clinical evaluation.

In recent years, the potential genes associated with STEMI and stable CAD have been obtained through microarray analysis of the peripheral blood of patients with AMI and the myocardium of mice [10–12]. However, integrated bioinformatics analysis is rarely employed in patients with DM and cardiovascular diseases. In this study, multiple gene datasets including GSE775, GSE19322, and GSE97494 were meta-analyzed to identify differentially expressed genes (DEGs) in the left ventricle (LV) myocardium between infarcted mice and SHAM mice. Multiple gene datasets including GSE3313, GSE10617, and GSE136948 were meta-analyzed to obtain DEGs of skeletal muscle from 11 diabetes and 10 wild-type mice. A Venn diagram was applied to obtain the overlapping DEGs, and further hub genes were obtained and validated to investigate the potential biomarkers for patients with DM and undergoing MI at the same time.

## 2. Methods

### 2.1. Microarray Data

Using the keywords “myocardial infarction” in “*Mus musculus,*” we found three GEO datasets, including GSE775, GSE19322, and GSE97494. There were 18 samples of LV myocardium between the LAD artery and the apex at 1 day after the operation, which were from 9 infarcted mice and 9 SHAM mice. Using the keywords “diabetes” in “*Mus musculus,*” we found three GEO datasets, including GSE3313, GSE10617, and GSE136948. There were 21 skeletal muscle samples from 11 DM and 10 wild-type mice (Table 1).

### 2.2. Identification of DEGs through Integrated Bioinformatics Analysis

To screen out DEGs, the series matrix files were downloaded and analyzed by NetworkAnalyst 3.0 (http://www.networkanalyst.ca) through Log2 transformation normalization. Utilizing Limma, a log2 (fold change) > 1 and a *p* value < 0.05 were applied as the cutoff criteria.

Cluster analysis and integrated bioinformatics analysis were used to visualize the interaction relationships of the DEGs in the DM and AMI datasets, respectively. Representative images, including heatmap, the gene cluster in the PCA loading score, and the chord diagram of the datasets, were visualized using NetworkAnalyst 3.0. The KEGG topology enrichment analysis, protein-protein Internet (PPI) topology analysis, and Gene Regulatory Networks TF-gene interactions analyses were also applied.

### 2.3. Enrichment Analysis of the Same Transcripts between DEGs of DM and AMI Datasets

The same transcripts between DEGs of DM and AMI datasets were identified, and Venn diagrams were drawn (http://bioinformatics.psb.ugent.be/webtools/Venn/). KEGG [13] and GO [14] analyses were performed using overrepresentation analysis methods in the Web-based Gene Set Analysis Toolkit (WebGestalt, http://www.webgestalt.org) and metascape (http://www.metascape.org). The KEGG topology enrichment and PPI topology analyses were also performed using NetworkAnalyst 3.0.

### 2.4. Prediction of Pivotal miRNAs and Construction of Gene-miRNA Interaction Network Analysis

Using miRWalk 2.0, the prediction of hub gene-targeted miRNAs was performed and validated using TargetScan and miRDB. To verify the accuracy of the results, the miRNA dataset GSE114695 and the predicted miRNAs were used to perform intersections. The final result obtained from the intersection was further processed using Cytoscape v 3.7.1.

### 2.5. MI Model Samples' Validation

Adult experimental C57Bl/6J male mice were purchased from Charles River (Beijing, China). Mice were maintained in a specific pathogen-free environment with free access to food and water and a 12/12 light-dark cycle. Protocols were approved by the Institute of Radiation Medicine, Chinese Academy of Medical Science, and conformed to the Guide for the Care and Use of Laboratory Animals.

Mice were fed a high-fat diet (HFD) for 3 months and administrated streptozotocin (STZ, i.p., 50 mg/kg in citrate buffer, S0130, Sigma), and glucose levels of blood obtained via mice tail prick were measured twice at 24-hour intervals. MI was induced in young mice (19–20 weeks). Briefly, the heart was manually exposed from the 4th intercostal space through inhalation of isoflurane (1.5–2%, MSS-3, England) and the left coronary artery was located, sutured, and ligated at a site approximately 3 mm from its origin, which induced approximately 50% ischemia of the left ventricle in mice. Infarction was considered successful following the visual appearance of pale discoloration and ST-elevation on an electrocardiogram. Sham-operated animals underwent the same procedure as the MI model without coronary artery ligation.

Cardiac function was evaluated using a Vevo 2100 System equipped with a 30 MHz transducer (FUJIFILM VisualSonics, Inc. Toronto, Canada) at 1 day or 1 week after surgery. The investigator was blinded to group assignment. Mice were anaesthetized by inhalation of isoflurane (1–1.5%, MSS-3, England) and moved to a warming plate that maintained the core body temperature. Heart function was detected through a two-dimensional parasternal long axis. The limb lead electrocardiogram (ECG) was also recorded, and the corresponding PR and QRS intervals of each group were measured and analyzed based on the ECG records of at least 100 beats. Left ventricular ejection fraction (LVEF, %) was measured using M-mode.

Total RNA of left ventricular samples from diabetes MI mice and SHAM mice was isolated using the RNeasy Mini Kit (Qiagen, Valencia, CA). cDNA was synthesized using the TaqMan reverse-transcription reagents (Applied Biosystems, Foster City, CA, USA). For qRT-PCR analysis, 50 ng of cDNA was used as a template in triplicate reactions for each primer pair, and the assay was performed using an iCycler iQ™ Real-Time PCR Detection System (Bio-Rad, Hercules, CA, USA) with the primers. The primer sequences of the hub genes used in qRT-PCR analysis are shown in Table 2.

For further validation, PIK3R1, SPNB2, and CRYAB levels in the left ventricular samples of the diabetes MI mice were determined using a mouse PIK3R1 ELISA kit (m18136521, mlbio, Shanghai, China), mouse SPNB2 ELISA kit (m18236541, mlbio,) and mouse CRYAB ELISA kit (m18695245, mlbio), respectively.

### 2.6. Human Blood Samples' Validation

For further validation, the blood samples of the patients with DM who did or did not experience MI were collected and the blood samples of the DM patients with AMI were collected twice (immediately admitted and immediately discharged). The inclusion criteria were as follows: (1) a history of DM of >5 years, (2) DM who did or did not experience AMI, and (3) whose laboratory examinations were completed. The exclusion criteria were as follows: (1) a history of nephropathy, especially induced by DM, (2) a history of hepatopathy, (3) a history of diabetic retinopathy, (4) a history of tumour, (5) cardiac arrest or extracorporeal cardiopulmonary resuscitation (ECPR), (6) multiple organ dysfunction syndrome (MODS) or irreversible brain damage, (7) within 3 months after surgery, (8) aortic insufficiency or aortic dissection, and (9) uncontrollable bleeding. The protocols were approved by the Tianjin Third Central Hospital.

The hub genes were determined using a human PIK3R1 ELISA kit (m18266954, mlbio), human SPNB2 ELISA kit (m18365412, mlbio), and human CRYAB ELISA kit (m18362595, mlbio), respectively. Clinical information and laboratory examinations, such as the level of cTnI and pro-BNP levels, were also collected.

Data are presented as mean ± standard deviation, median (Q1–Q3), or frequency (percentage). Statistical analyses were performed using SPSS 23.0. The Shapiro–Wilk normality test and Weltch's *t*-test (two groups) were used and the Spearman correlation analysis was applied to the LVEF and protein levels of the screened hub genes. Correlation analysis was also performed to validate the effects of the hub genes on patients with DM and undergoing MI. Statistically significance was set as *p* < 0.05.

## 3. Results

### 3.1. Identification of DEGs and Integrated Bioinformatics Analysis

**Figure 1:**
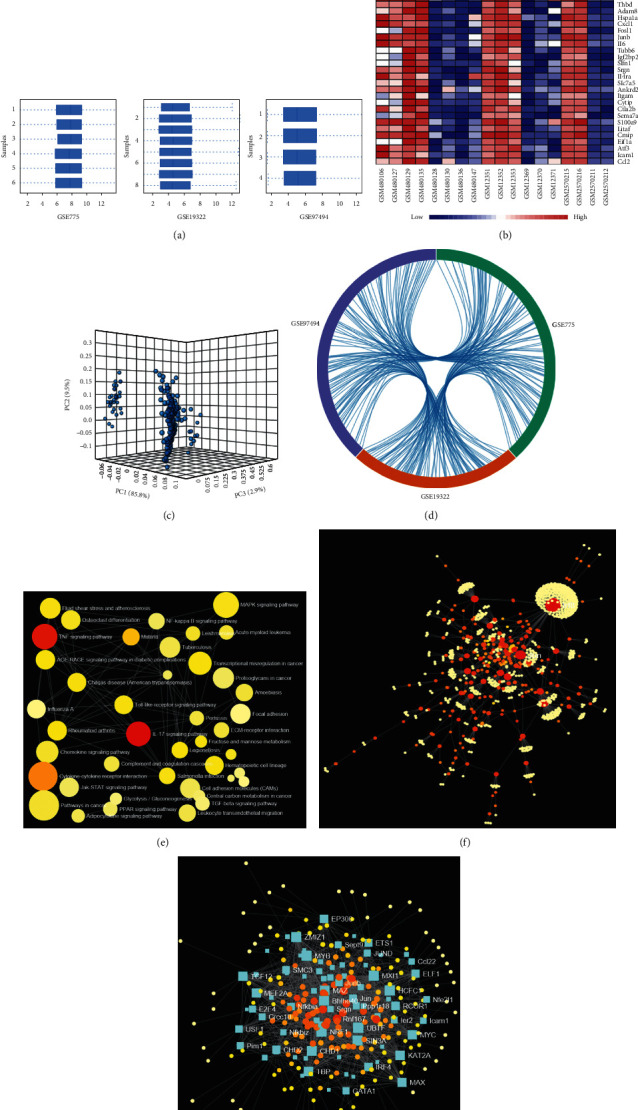
Integrated bioinformatics analysis of AMI GEO datasets. (a) The normalization of three AMI datasets was applied through Log2 transformation. (b) Heatmap of three datasets demonstrated distinguished features between AMI and SHAM heart samples. (c) The gene cluster in PCA loading score. (d) The chord diagram of three AMI GEO datasets. (e) The KEGG topology enrichment analysis after integrated bioinformatics analysis. (f) The PPI topology analysis after integrated bioinformatics analysis. (g) The Gene Regulatory Networks TF-gene interactions analysis after integrated bioinformatics analysis.

The hree datasets GSE775, GSE19322, and GSE97494 were utilized for the meta-analysis of MI datasets. Using the Limma package, 353 DEGs were identified from the meta-analyses (Figure 1; Fig. S1).

**Figure 2:**
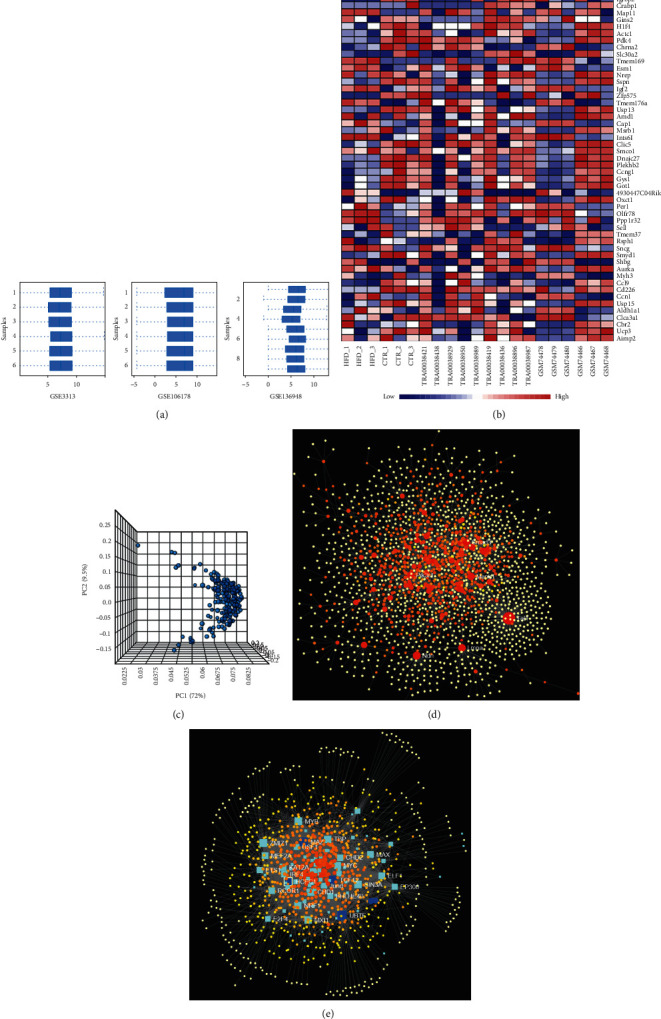
Integrated bioinformatics analysis of Diabetes GEO datasets. (a) The normalization of three diabetes datasets was applied through Log2 transformation. (b) Heatmap of three datasets demonstrated distinguished features between diabetes and WT skeletal muscle samples. (c) The gene cluster in PCA loading score. (d) The PPI topology analysis after integrated bioinformatics analysis. (e) The Gene Regulatory Networks TF-gene interactions analysis after integrated bioinformatics analysis.

The three datasets GSE3313, GSE10617, and GSE136948 were utilized for the meta-analysis of the DM datasets. Using the Limma package, 1937 DEGs were obtained from the meta-analyses (Figure 2; Fig. S1).

**Figure 3:**
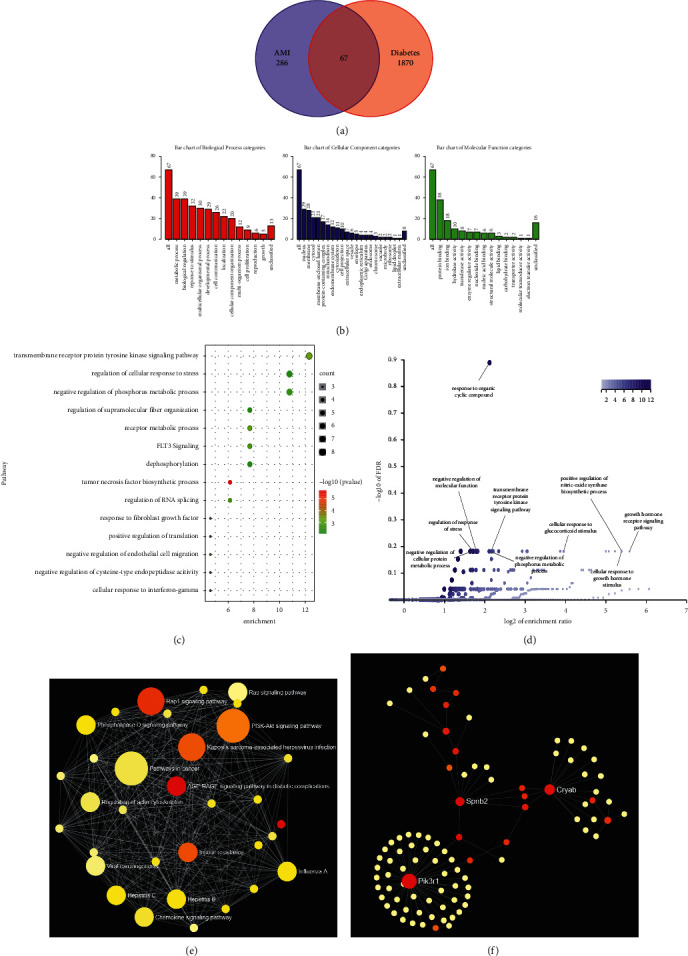
Differentially expressed transcripts among diabetes DEGs and AMI DEGs. (a) Venn diagram demonstrated 67 same expressed transcripts were screened among meta diabetes DEGs and meta AMI DEGs after integrated bioinformatics analysis. (b) GO slim of the 67 DEGs using WebGestalt. (c) The bubble plot of GO analysis about the 67 DEGs using Metascape. (d) The volcano plot of enrichment pathways after GO analysis using WebGestalt. (e) The KEGG topology enrichment analysis of the 67 DEGs using NetworkAnalyst. (f) The PPI topology analysis of the 67 DEGs using NetworkAnalyst.

Venn diagrams were used to obtain the overlapping DEGs between MI and DM and 67 DEGs were screened out, which may be potential biomarkers for patients with DM and undergoing MI (Figure 3(a); Table 3).

### 3.2. Enrichment Analysis of DEGs

GO slim and enrichment analyses were performed, which showed enrichment in 10 pathways, such as response to organic cyclic compound, regulation of response to stress, and negative regulation of molecular function (Figures 3(b)–3(d) and S2; Table 4).

KEGG pathway analysis was also performed, which were mainly enriched in 13 pathways, for instance, PI3K-Akt signaling pathway and Rap1 signaling pathway (Figure 3(e)).

### 3.3. Detection of the Key Genes in STEMI and Stable CAD

A PPI network analysis was applied to detect hub genes in patients with DM and undergoing MI. Three hub genes were screened out, including PIK3R1, SPNB2 (also called SPTBN1), and CRYAB (Figure 3(f)).

### 3.4. Further miRNA Mining and Interaction Network Analysis

**Figure 4:**
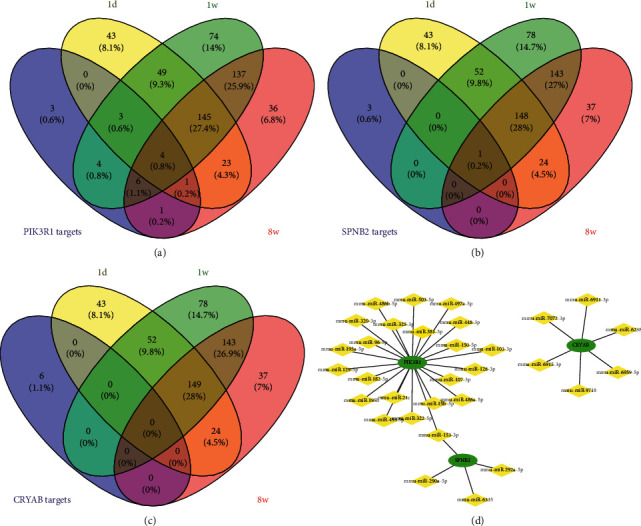
miRNA-hub genes network. Venn diagram among predicted miRNAs which bind the 5′UTR, CDS, and 3′UTR of hub genes PIK3R1 (a), SPNB2 (b), or CRYAB (c) and miRNAs which were screened at 1d, 1w, and 8w after MI in GSE114695 for validation. (d) The network of screened hub genes and miRNAs after validation.

To investigate the key genes and their interactive miRNAs, the prediction of hub gene-targeted miRNAs was performed and validated using miRWalk, TargetScan, and miRDB. To verify the accuracy of the results, the miRNA dataset in GSE114695 and the predicted miRNAs were used to perform intersections, and Cytoscape was used to draw the interaction network (Figure 4).

### 3.5. Validations of the Screened Hub Genes in the Mouse MI Model

**Figure 5:**
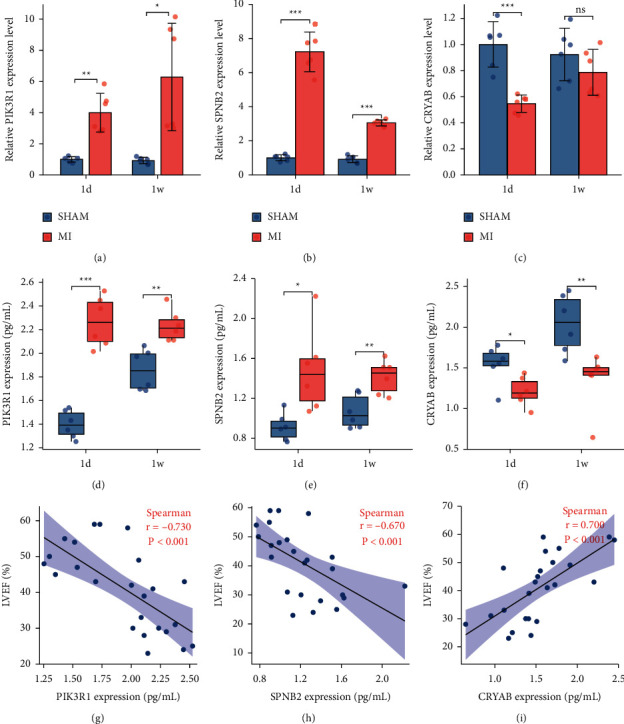
Validation of screened hub genes in mice MI model. The relative mRNA expression level of PIK3R1 (a), SPNB2 (b), and CRYAB (c) at 1d and 1w after MI using qPCR analysis. The protein expression levels of left ventricle at 1d and 1w after MI utilizing ELISA, including PIK3R1 (d), SPNB2 (e), and CRYAB (f). The expression levels of proteins, including PIK3R1 (g) and SPNB2 (h), were demonstrated to be negatively correlated to LVEF. The expression levels of protein CRYAB (i) were demonstrated to be positively correlated to LVEF. ^*∗*^*p* < 0.05,  ^*∗∗*^*p* < 0.01,  ^*∗∗∗*^*p* < 0.001; ns, not significant.

To validate the findings, a diabetes MI mouse model was constructed, and qPCR and ELISA of left ventricular samples were utilized. The mRNA and protein expression of PIK3R1 and SPNB2 increased in the diabetic MI group compared to the SHAM group, whereas the mRNA and protein expression of CRYAB decreased in the diabetes MI mice (Figures 5(a)–5(f)). The correlation between the protein levels of the three hub genes and LVEF was also applied, suggesting that the three hub genes may be diagnostic and therapeutic targets for patients with DM and undergoing MI at the same time (Figures 5(g)–5(i)).

### 3.6. Validation of the Screened Hub Genes in Humans

**Figure 6:**
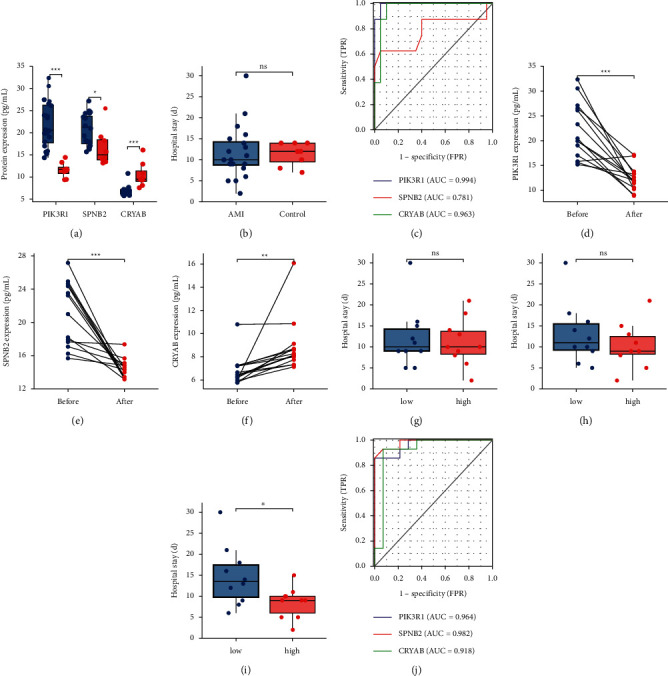
Validation of screened hub genes in human blood samples. (a) The protein expression levels of human blood samples between diabetes patients with and without AMI utilizing ELISA analysis, including PIK3R1, SPNB2, and CRYAB. (b) There is no significant difference in the hospital stay between diabetes patients with and without AMI. (c) The AUCs of three screened proteins of human blood samples between diabetes patients with and without AMI, including PIK3R1, SPNB2, and CRYAB, suggesting the three hub genes can be the diagnostic biomarkers for diabetic patients undergoing AMI. The protein expression levels of diabetic AMI patients' blood samples between before and after treatment utilizing ELISA analysis, including PIK3R1 (d), SPNB2 (e), and CRYAB (f). (g–h) There is no significant difference in the hospital stays between the diabetic AMI patients with high PIK3R1 and SPNB2 expression level and with low PIK3R1 and SPNB2 expression level. (i) The diabetic AMI patients with low PIK3R1 and SPNB2 expression level had the longer hospital stays compared to those with high PIK3R1 and SPNB2 expression level. (j) The AUCs of three screened proteins of diabetic AMI patients' blood samples before and after treatment, including PIK3R1, SPNB2, and CRYAB, suggesting the three hub genes can be the prognostic biomarkers for patients with diabetes and undergoing AMI. ^*∗*^*p* < 0.05,  ^*∗∗*^*p* < 0.01,  ^*∗∗∗*^*p* < 0.001; ns, not significant.

To further validate whether the three hub genes may be diagnostic and therapeutic targets for patients with DM and undergoing MI, blood samples from patients with DM who underwent STEMI or not were collected and subjected to ELISA. There was no significant difference in laboratory examinations between DM patients with and without AMI or between DM patients with AMI before and after treatment, including age, BMI, and uric acid level (Tables S1 and S1). PIK3R1 and SPNB2 were highly expressed at 1 day after AMI compared to the control group, whereas CRYAB was expressed at low levels. ROC analysis was performed to investigate the diagnostic abilities. The areas under the curve (AUC) of the three proteins were 0.994, 0.781, and 0.963, respectively (Figures 6(a)–6(c)). Besides, the PIK3R1 and SPNB2 levels of most AMI patients decreased after treatment, whereas the CRYAB level of most AMI patients increased after treatment. Patients with high CRYAB expression had a shorter hospital stay than those with low CRYAB expression. The AUCs of the three protein levels before and after treatment were 0.964, 0.982, and 0.918, suggesting that PIK3R1, SPNB2, and CRYAB may be diagnostic and prognostic biomarkers for patients with DM and undergoing AMI (Figures 6(d)–6(j)).

**Figure 7:**
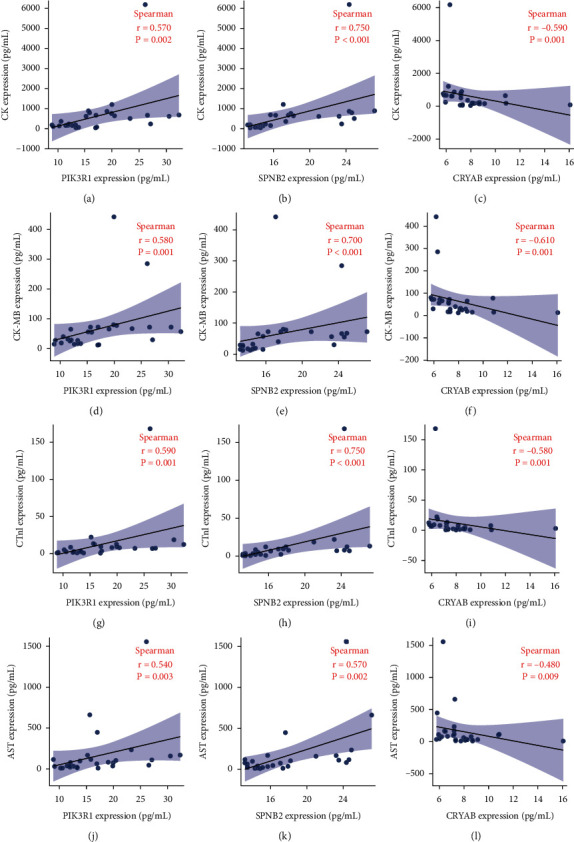
The correlation analysis of the screened hub genes and clinical laboratory examination. The expression levels of proteins, including PIK3R1 (a) and SPNB2 (b), were demonstrated to be negatively correlated to CK expression, while the CRYAB (c) expression was demonstrated to be positively correlated to CK expression. The expression levels of proteins, including PIK3R1 (d) and SPNB2 (e), were demonstrated to be negatively correlated to CK-MB expression, while the CRYAB (f) expression was demonstrated to be positively correlated to CK-MB expression. The expression levels of proteins, including PIK3R1 (g) and SPNB2 (h), were demonstrated to be negatively correlated to cTnI expression, while the CRYAB (i) expression was demonstrated to be positively correlated to cTnI expression. The expression levels of proteins, including PIK3R1 (j) and SPNB2 (k), were demonstrated to be negatively correlated to AST expression, while the CRYAB (l) expression was demonstrated to be positively correlated to AST expression.

To investigate the association between the three hub genes and the significantly different laboratory examinations, correlation analysis was utilized, and it was found that the PIK3R1 and SPNB2 levels were positively correlated to the expression of CK, CK-MB, CTnI, and AST, whereas the CRYAB level was negatively correlated to the protein expression (Figure 7). However, the expression of these three proteins did not correlate to BNP and ALT expression (Figure S1).

## 4. Discussion

Previous studies have demonstrated the etiology of stable CAD and MI; however, predictive biomarkers and treatment targets are still limited. The mortality and morbidity of patients with DM and undergoing AMI are still increasing, and the increased in-hospital mortality and morbidity of DM patients with STEMI is mainly driven by their underlying cardiorenal dysfunction [6, 15]. Therefore, the integrated bioinformatics analysis and screening of hub genes may be helpful for patients with DM and cardiovascular disease.

In this study, the datasets including GSE775, GSE19322, and GSE97494 were meta-analyzed to obtain DEGs of LV myocardium between infarcted mice and SHAM mice. Multiple gene datasets including GSE3313, GSE10617, and GSE136948 were used to screen new potential biomarkers of skeletal muscle from 11 diabetes and 10 wild-type mice. A Venn diagram was applied to obtain the overlapping DEGs, and further hub genes were obtained and validated to investigate the potential biomarkers for patients with DM and undergoing MI. The LV samples of the mouse MI model and human blood samples were collected and analyzed for the further validation of the screened hub genes. Thus, PIK3R1, SPNB2, and CRYAB were demonstrated to serve as novel biomarkers for patients with DM and AMI.

PIK3R1 and SPNB2 were highly expressed at 1 day after AMI compared to the control group, whereas CRYAB was expressed at low levels. Patients with high CRYAB expression had a shorter hospital stay than that of patients with low CRYAB expression. In addition, PIK3R1 and SPNB2 levels were positively correlated to the expression of CK, CK-MB, CTnI, and AST, while the CRYAB level was negatively correlated to. The AUCs of the three protein levels before and after treatment were 0.964, 0.982, and 0.918, suggesting that PIK3R1, SPNB2, and CRYAB may be diagnostic and prognostic biomarkers for AMI patients with DM.

The mRNA and protein expression of PIK3R1 and SPNB2 increased in AMI patients with DM, whereas that of CRYAB decreased. Phosphoinositide-3-kinase regulatory subunit 1 (PIK3R1) can be enhanced by CapG, an additional oncogenic signaling mutation that leads to increased PI3K/Akt activation [16–18]. Somatic PIK3R1 variation can also be attributed to genetic variation in the PI3K-AKT pathway and can induce vascular malformations and overgrowth [19]. The PI3K*δ* enzyme complex, including PIK3CD, PIK3R1, or phosphatase and tensin homolog (PTEN), is primarily present in the immune system and comprises a catalytic (p110*δ*) and regulatory (p85*α*) subunit, and both overactivation and underactivation of PI3K*δ* lead to impaired and dysregulated immunity, for instance, B-cell lymphomas [20, 21]. In this study, PIK3R1 may also play a critical role in AMI progression in patients with DM, which may serve as a novel biomarker and therapeutic target for diabetic cardiomyopathy. Spectrin beta 2 (SPNB2), an essential component of the red blood cell membrane skeleton, is tissue-specific with the exception of the 7.2-kb transcript being unique to heart and skeletal muscle tissues [22]. SPNB2 is demonstrated to be highly expressed in the adipogenic process [23] and can also be a TGF-*β* adapter that regulates notch signaling and SOX9 expression in esophageal adenocarcinoma [24]. Besides, SPNB2 deletion in mice contributes to the inactivation of TGF-*β*/Smad signaling and prevents proper development of the heart in association with the disintegration of dystrophin structure and markedly reduced survival [25]. In our study, SPNB2 expression was higher in DM patients with AMI than in those without AMI, which may be due to its regulation of TGF-*β* signaling pathway. Alpha B-crystallin (CRYAB) can retain fundamental functions in striated muscle during physiological or pathological modifications and p-38MAPK-mediated phosphorylation can promote its interaction with the myofibrillar components, such as *β*-actin, desmin, and filamin, which can activate actin cytoskeleton-mediated vascular remodeling [26, 27]. An increased stress response is related to the increased expression of proteins in titin-based mechanotransduction, such as CRYAB [28]. Besides, inhibition of Ube2v1 decreases CryABR120G (a mutant CRYAB in cardiomyocytes) induced aggregate formation through enhanced ubiquitin proteasome system performance [29, 30]. Phosphodiesterase 1 can reduce myocardial misfolded CryAB and increase PKA-mediated proteasome phosphorylation, thereby treating heart failure with preserved ejection fraction caused by CryABR120G, which may provide a novel therapy for cardiac diseases with increased proteotoxic stress [31]. In this study, CRYAB expression decreased in AMI patients with DM, which may be associated with CryABR120G expression and could be a prognostic biomarker for cardiac function after stress.

There are some limitations to this study. First, three hub genes were validated using a mouse MI model and human blood samples. There may be some false negatives due to the enrichment methods and validation methods. More researches are needed to validate the effects of neighboring genes on cardiac function. Second, we aimed to investigate the potential targets for DM patients with AMI using integrated bioinformatics analysis. Therefore, we could only discuss a few significant genes and their neighbors in this study, and we hope to explore the others in the future. Lastly, while the sample sizes of the datasets were not large, after the calculation of sample sizes, they still met the requirements for further enrichment analysis and other statistical methods.

## 5. Conclusions

In conclusion, our study provides an integrated bioinformatics analysis of DM patients with AMI. The screened hub genes, PIK3R1, SPNB2, and CRYAB, have been validated as credible molecular biomarkers using the mouse MI model and human blood samples, which may provide a novel therapy for cardiac diseases with increased proteotoxic stress.

## Figures and Tables

**Table 1:** Details for GEO data.

Reference	Sample	GEO	Platform	Diseases	Normal
Schnike et al. (2003)	LV myocardium	GSE775	GPL81	3 AMI	3 SHAM
Hunt et al. (2010)	LV myocardium	GSE19322	GPL339	4 AMI	4 SHAM
Chikata et al. (2017)	LV myocardium	GSE97494	GPL6246	2 AMI	2 SHAM
Schiekofer et al. (2014)	Skeletal muscle	GSE3313	GPL1261	3 diabetes	3 WT
Spallotta et al. (2018)	Skeletal muscle	GSE106178	GPL19057	3 diabetes	3 WT
O'Neill et al. (2019)	Skeletal muscle	GSE136948	GPL17021	5 diabetes	4 WT

GEO, Gene Expression Omnibus; AMI, acute myocardial infarction; LV, left ventricular; WT, wild type.

**Table 2:** Primer Sequences of the hub genes used in qRT-PCR analysis.

Pimer name	Primer sequence (5′–3′)
Pik3r1	F: AAGAAGTTGAACGAGTGGTTGG
	R: GCCCTGTTTACTGCTCTCCC
Sptbn1	F: GCCATTGAAACAGACATTG
	R: CCCACAGGCGTATAACATTG
Cryab	F: TCTCTACAGCCACTTCCCTG
	R: TGTCCTTCTCCATACGCATC

**Table 3:** The same expressed transcripts among meta diabetes DEGs and meta AMI DEGs after integrated bioinformatics analysis.

DEGs	Gene names
Upregulated (32 DEGs)	Dhrs7b, Isca1, smpdl3a, Trappc2l, Nudt19, Dnaja3, Pik3r1, Ccdc90b, Clip1, Timm23, Ablim1, Gpcpd1, Spryd4, Mrpl18, Uqcc1, Ankrd40, slc12a7, Prpf19, Kif16b, Mrpl50, Psme4, Fem1a, Zmynd11, Dbt, Rnf13, Eln, Atad1, Gys1, Spnb2, Nampt, Echs1, slc35e1
Downregulated (35 DEGs)	Dusp6, Ier2, Nfkbia, Rrp1b, Thbd, Fmr1, Ccnl2, Jak2, Btg1, Srsf11, Ctla2b, Klf6, Ifit1, Errfi1, Ptpn12, Akap12, Cryab, Mcam, slc20a1, Spata13, Wfdc21, Thbs1, Ankrd13a, Angpt2, Ppp1r15b, Ralb, Gna13, Tle3, Ppp1r18, Tm4sf1, Ugdh, Glrx, Rbms1, Stc1, Nfkbiz

The three screened hub genes were labeled red and blue in the table.

**Table 4:** The Top10 GO analysis of the same expressed transcripts among meta diabetes DEGs and meta AMI DEGs after integrated bioinformatics analysis.

Gene set	Description	Size	Expect	Ratio	P value	Fdr
GO:0014070	Response to organic cyclic compound	926	2.7436	4.3739	0.000013919	0.12998
GO:0080134	Regulation of response to stress	1227	3.6354	3.3009	0.00021096	0.65917
GO:0044092	Negative regulation of molecular function	1062	3.1465	3.4959	0.00024682	0.65917
GO:0032269	Negative regulation of cellular protein metabolic process	1017	3.0132	3.3188	0.00073479	0.65917
GO:0060396	growth hormone receptor signaling pathway	14	0.041479	48.217	0.00076733	0.65917
GO:0007169	Transmembrane receptor protein tyrosine kinase signaling pathway	524	1.5525	4.5088	0.00087297	0.65917
GO:0071385	Cellular response to glucocorticoid stimulus	65	0.19258	15.578	0.00094632	0.65917
GO:0051770	Positive regulation of nitric-oxide synthase biosynthetic process	16	0.047405	42.19	0.001008	0.65917
GO:0071378	Cellular response to growth hormone stimulus	16	0.047405	42.19	0.001008	0.65917
GO:0010563	Negative regulation of phosphorus metabolic process	544	1.6118	4.3431	0.0010856	0.65917

## Data Availability

The datasets can be investigated in GEO datasets, NCBI.

## Abbreviations

AMI: Acute myocardial infarction
STEMI: ST-elevated myocardial infarction
DM: Diabetes mellitus
ESC: European Society of Cardiology
DEGs: Differentially expressed genes
LV: Left ventricle
CAD: Coronary artery disease
STEMI: ST-elevated myocardial infarction
GO: Gene ontology
KEGG: Kyoto Encyclopedia of Genes and Genomes
PPI: Protein-protein Internet
STZ: Streptozotocin
ECG: Electrocardiogram
LVEF: Left ventricular ejection fraction
qRT-PCR: Quantitative real-time reverse-transcription polymerase chain reaction
ELISA: Enzyme-linked immunosorbent assay
AUC: Area under curve
PIK3R1: Phosphoinositide-3-kinase regulatory subunit 1
PTEN: Phosphatase and tensin homolog
SPNB2: Spectrin beta 2
CRYAB: Alpha B-crystallin
ECPR: Extracorporeal cardiopulmonary resuscitation
MODS: Multiple organ dysfunction syndrome.

## Supplementary Materials

Supplementary Materials Supplementary Figure 1. The volcano plot of meta diabetes DEGs (left) and meta AMI DEGs (right) after integrated bioinformatics analysis. Supplementary Figure 2. The GO analysis of the 67 screened DEGs. (a) The GO topology analysis of the 67 DEGs using Metascape. (b) The GO-Dags of the 67 screened DEGs using WebGestalt. Supplementary Figure 3. The correlation analysis of the screened hub genes and clinical laboratory examination. The expression levels of PIK3R1 (a), SPNB2 (b), and CRYAB (c) were not correlated to BNP expression. The expression levels of PIK3R1 (d), SPNB2 (e), and CRYAB (f) were not correlated to ALT expression. .
