# Supplementary material for: PIK3R1, SPNB2, and CRYAB as Potential Biomarkers for Patients with Diabetes and Developing Acute Myocardial Infarction
Source: Int J Endocrinol. 2021 Nov 30;2021:2267736. doi: 10.1155/2021/2267736 (PMC8651423; doi:10.1155/2021/2267736)
Supplement: Supplementary Materials — Supplementary Figure 1. The volcano plot of meta diabetes DEGs (left) and meta AMI DEGs (right) after integrated bioinformatics analysis. Supplementary Figure 2. The GO analysis of the 67 screened DEGs. (a) The GO topology analysis of the 67 DEGs using Metascape. (b) The GO-Dags of the 67 screened DEGs using WebGestalt. Supplementary Figure 3. The correlation analysis of the screened hub genes and clinical laboratory examination. The expression levels of PIK3R1 (a), SPNB2 (b), and CRYAB (c) were not correlated to BNP expression. The expression levels of PIK3R1 (d), SPNB2 (e), and CRYAB (f) were not correlated to ALT expression. . [file 2267736.f1.zip › 2267736.f1/Supplementary Table.docx]

Supplementary Table 1. Comparisons of demographic data and laboratory parameters among diabetes patients with or without AMI.

| Characteristic | AMI patients | Control | *P* | method |
| --- | --- | --- | --- | --- |
|  | N=20 | N=8 |  |  |
| Male, n (%) | 15 (75%) | 6 (75%) | 1.000 | Fisher.test |
| Age, year | 57.65 ± 12.91 | 61.5 ± 8.38 | 0.445 | T test |
| BMI, kg/m^2^ | 25.59 ± 3.78 | 27.71 ± 2.71 | 0.162 | T test |
| SBP, mmHg | 124 ± 27.62 | 149.88 ± 17.15 | 0.021 | T test |
| DBP, mmHg | 68.7 ± 10.65 | 82.12 ± 5.41 | 0.002 | T test |
| Smoke, n (%) | 11 (55%) | 5 (62.5%) | 1.000 | Fisher.test |
| UGLU, n (%) |  |  | 0.115 | Chisq.test |
| 0 | 3 (15%) | 0 (0%) |  |  |
| 1 | 2 (10%) | 0 (0%) |  |  |
| 2 | 0 (0%) | 2 (25%) |  |  |
| 3 | 1 (5%) | 0 (0%) |  |  |
| 3+/4 | 14 (70%) | 6 (75%) |  |  |
| [Urine](javascript:;) [protein](javascript:;), n (%) |  |  | 0.806 | Chisq.test |
| 0 | 14 (70%) | 5 (62.5%) |  |  |
| 1 | 3 (15%) | 2 (25%) |  |  |
| 2 | 1 (5%) | 0 (0%) |  |  |
| 3 | 1 (5%) | 1 (12.5%) |  |  |
| 3+/4 | 1 (5%) | 0 (0%) |  |  |
| TG, mmol/L | 1.31 (0.99, 2.21) | 2.04 (1.86, 3.14) | 0.050 | Wilcoxon |
| LDL, mmol/L | 2.84 ± 1.04 | 2.2 ± 0.72 | 0.125 | T test |
| HDL, mmol/L | 0.97 (0.87, 1.06) | 0.88 (0.8, 1.01) | 0.333 | Wilcoxon |
| TC, mmol/L | 4.33 ± 1.33 | 4.26 ± 0.83 | 0.889 | T test |
| CK, ng/ml | 673 (630.25, 800) | 84 (76, 95.5) | < 0.001 | Wilcoxon |
| CK-MB, ng/ml | 72 (56.75, 78.5) | 13.5 (13, 14) | < 0.001 | Wilcoxon |
| CTnI, ng/ml | 11.4 (8.73, 13.33) | 0.33 (0.2, 0.34) | < 0.001 | Wilcoxon |
| BNP, pg/ml | 118 (106.75, 181.25) | 34.7 (24.9, 45.08) | < 0.001 | Wilcoxon |
| UA, mmol/L | 319.2 ± 112.63 | 296.5 ± 108.79 | 0.667 | T test |
| Cr, µmol/L | 74 (64.75, 85.75) | 75 (57.75, 79.75) | 0.760 | Wilcoxon |
| FVBG, mmol/L | 7.76 (6.84, 11.96) | 8.25 (7.42, 11.75) | 0.741 | Wilcoxon |
| G-HB, mmol/L | 8.96 ± 2.29 | 10.04 ± 2.9 | 0.309 | T test |
| Alb, g/L | 38.57 ± 4.49 | 38.92 ± 4.06 | 0.848 | T test |
| β2-MG, mg/L | 24.2 ± 4.75 | 22.7 ± 2.78 | 0.413 | T test |
| ALP, U/L | 67.85 ± 16.39 | 80.25 ± 18.59 | 0.093 | T test |
| ALT, U/L | 30 (20, 53.5) | 13 (12.25, 15.75) | 0.002 | Wilcoxon |
| AST, U/L | 114 (67.75, 186.25) | 13.5 (11, 14.25) | < 0.001 | Wilcoxon |

Data are presented as mean ± SD, median (Q1–Q3), or frequency (percentage). AMI patients indicate diabetes patients with AMI. Control indicate diabetes patients without AMI.

BMI indicates body mass index; SBP: systole blood pressure; DBP: diastole blood pressure; UGLU: urine glucose; TG: triglyceride; HDL: high-density lipoprotein; LDL: low-density lipoprotein; TC: total cholesterol; CK, creatine kinase; CK-MB, creatine kinase MB; CTnI, cardiac troponin I; BNP, B-type natriuretic peptide; UA: uric acid; Cr: creatinine; FVBG: Fasting venous blood glucose; G-HB: Glycosylated hemoglobin; Alb: albumin; β2-MG: β2-microglobulin; ALP: alkaline phosphatase; ALT: glutamic-pyruvic transaminase; AST: glutamic oxalacetic transaminase.

Red labelled, *P* < 0.05.

Supplementary Table 2. Comparisons of laboratory parameters of diabetes patients with AMI before and after treatment.

| Characteristic | before | after | *P* | method |
| --- | --- | --- | --- | --- |
| UGLU, n (%) |  |  | 0.204 | Chisq.test |
| 0 | 3 (15%) | 4 (20%) |  |  |
| 1 | 2 (10%) | 2 (10%) |  |  |
| 2 | 0 (0%) | 4 (20%) |  |  |
| 2+ | 1 (5%) | 1 (5%) |  |  |
| 3 | 14 (70%) | 9 (45%) |  |  |
| 3+/4 | 0 (0%) | 0 (0%) |  |  |
| Urine protein, n (%) |  |  | 0.558 | Chisq.test |
| 0 | 14 (70%) | 14 (70%) |  |  |
| 1 | 3 (15%) | 3 (15%) |  |  |
| 2 | 1 (5%) | 3 (15%) |  |  |
| 3 | 1 (5%) | 0 (0%) |  |  |
| 3+/4 | 1 (5%) | 0 (0%) |  |  |
| CK, ng/ml | 673 (630.25, 800) | 136.5 (82.75, 184.5) | < 0.001 | Wilcoxon |
| CK-MB, ng/ml | 72 (56.75, 78.5) | 18 (15, 28.25) | < 0.001 | Wilcoxon |
| CTnI, ng/ml | 11.4 (8.73, 13.33) | 2.34 (1.27, 3.26) | < 0.001 | Wilcoxon |
| BNP, pg/ml | 118 (106.75, 181.25) | 155 (109.75, 203.5) | 0.402 | Wilcoxon |
| Cr, µmol/L | 74 (64.75, 85.75) | 82.5 (75.25, 90) | 0.159 | Wilcoxon |
| FVBG, mmol/L | 7.76 (6.84, 11.96) | 7.62 (6.56, 10.11) | 0.433 | Wilcoxon |
| Alb, g/L | 38.57 ± 4.49 | 37.27 ± 4.39 | 0.360 | T test |
| β2-MG, mg/L | 24.2 ± 4.75 | 25.24 ± 3.82 | 0.450 | T test |
| ALP, U/L | 67.85 ± 16.39 | 69.2 ± 15.24 | 0.789 | T test |
| ALT, U/L | 30 (20, 53.5) | 28 (20.5, 41.25) | 0.534 | Wilcoxon |
| AST, U/L | 114 (67.75, 186.25) | 32.5 (20.25, 57.75) | < 0.001 | Wilcoxon |

Data are presented as mean ± SD, median (Q1–Q3), or frequency (percentage).

UGLU indicates urine glucose; CK, creatine kinase; CK-MB, creatine kinase MB; CTnI, cardiac troponin I; BNP, B-type natriuretic peptide; Cr: creatinine; FVBG: Fasting venous blood glucose; Alb: albumin; β2-MG: β2-microglobulin; ALP: alkaline phosphatase; ALT: glutamic-pyruvic transaminase; AST: glutamic oxalacetic transaminase.

Red labelled, *P* < 0.05.
