# Supplementary figures and images for: PIK3R1, SPNB2, and CRYAB as Potential Biomarkers for Patients with Diabetes and Developing Acute Myocardial Infarction
Source: Int J Endocrinol. 2021 Nov 30;2021:2267736. doi: 10.1155/2021/2267736 (PMC8651423; doi:10.1155/2021/2267736)

**AMI**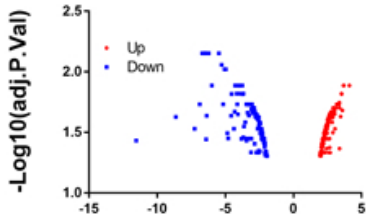**CombinedES****Diabetes**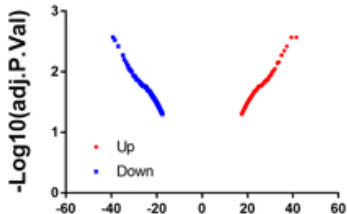**CombinedTstat**

Supplement: Supplementary Materials — Supplementary Figure 1. The volcano plot of meta diabetes DEGs (left) and meta AMI DEGs (right) after integrated bioinformatics analysis. Supplementary Figure 2. The GO analysis of the 67 screened DEGs. (a) The GO topology analysis of the 67 DEGs using Metascape. (b) The GO-Dags of the 67 screened DEGs using WebGestalt. Supplementary Figure 3. The correlation analysis of the screened hub genes and clinical laboratory examination. The expression levels of PIK3R1 (a), SPNB2 (b), and CRYAB (c) were not correlated to BNP expression. The expression levels of PIK3R1 (d), SPNB2 (e), and CRYAB (f) were not correlated to ALT expression. . [file 2267736.f1.zip › 2267736.f1/Supplementary Figure 1.pdf]

A

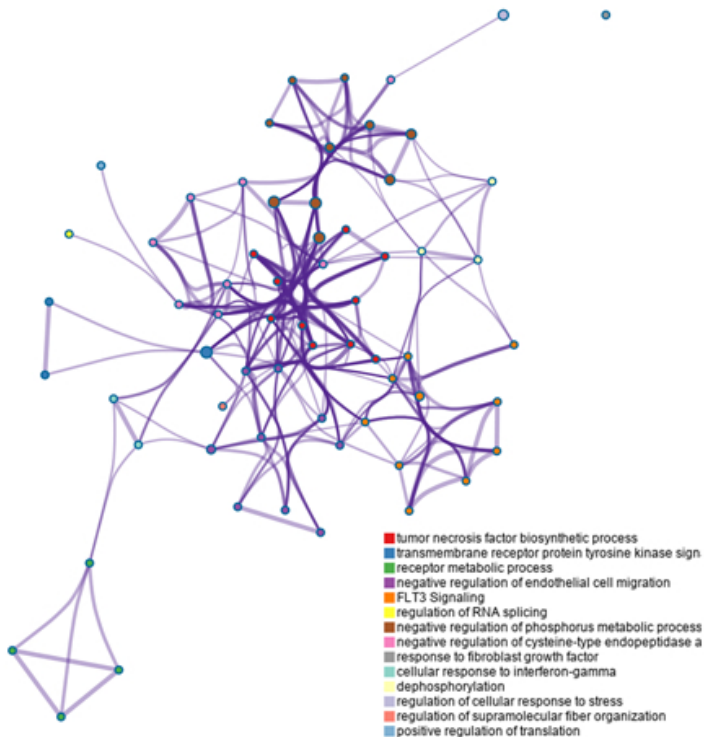

B

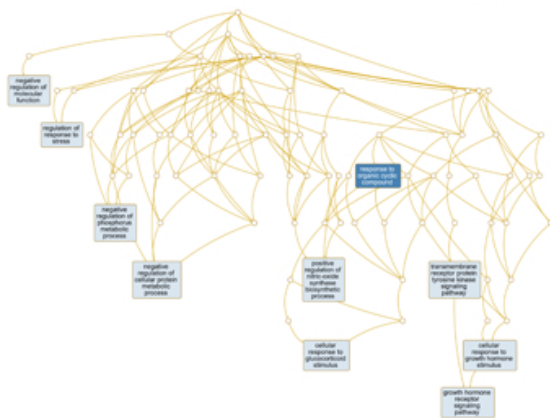

Supplement: Supplementary Materials — Supplementary Figure 1. The volcano plot of meta diabetes DEGs (left) and meta AMI DEGs (right) after integrated bioinformatics analysis. Supplementary Figure 2. The GO analysis of the 67 screened DEGs. (a) The GO topology analysis of the 67 DEGs using Metascape. (b) The GO-Dags of the 67 screened DEGs using WebGestalt. Supplementary Figure 3. The correlation analysis of the screened hub genes and clinical laboratory examination. The expression levels of PIK3R1 (a), SPNB2 (b), and CRYAB (c) were not correlated to BNP expression. The expression levels of PIK3R1 (d), SPNB2 (e), and CRYAB (f) were not correlated to ALT expression. . [file 2267736.f1.zip › 2267736.f1/Supplementary Figure 2.pdf]

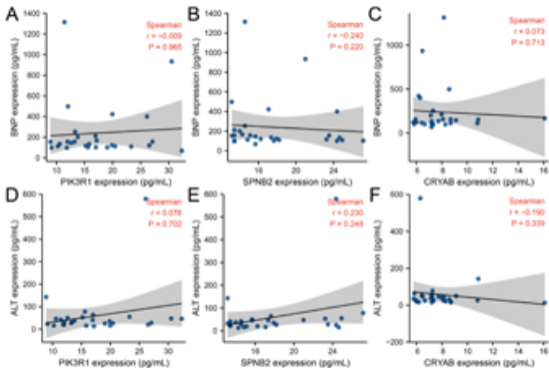

Supplement: Supplementary Materials — Supplementary Figure 1. The volcano plot of meta diabetes DEGs (left) and meta AMI DEGs (right) after integrated bioinformatics analysis. Supplementary Figure 2. The GO analysis of the 67 screened DEGs. (a) The GO topology analysis of the 67 DEGs using Metascape. (b) The GO-Dags of the 67 screened DEGs using WebGestalt. Supplementary Figure 3. The correlation analysis of the screened hub genes and clinical laboratory examination. The expression levels of PIK3R1 (a), SPNB2 (b), and CRYAB (c) were not correlated to BNP expression. The expression levels of PIK3R1 (d), SPNB2 (e), and CRYAB (f) were not correlated to ALT expression. . [file 2267736.f1.zip › 2267736.f1/Supplementary Figure 3.pdf]
